# First Brazilian datathon in critical care

**DOI:** 10.5935/0103-507X.20180006

**Published:** 2018

**Authors:** Ary Serpa Neto, Guillaume Kugener, Lucas Bulgarelli, Roberto Rabello Filho, Miguel Ángel Armengol de la Hoz, Alistair EW Johnson, Kenneth E Paik, Felipe Torres, Chen Xie, Edson Amaro Júnior, Leonardo José Rolim Ferraz, Leo Anthony Celi, Rodrigo Octavio Deliberato

**Affiliations:** 1 Department of Critical Care Medicine, Hospital Israelita Albert Einstein - São Paulo (SP), Brazil.; 2 Department of Intensive Care, Academic Medical Center, University of Amsterdam - Amsterdam, The Netherlands.; 3 Laboratory for Computational Physiology, Harvard-MIT Health Sciences & Technology, MIT - Cambridge, Massachusetts, United States.; 4 Big Data Analytics Group, Hospital Israelita Albert Einstein - São Paulo (SP), Brazil.; 5 Department of Anesthesia, Critical Care and Pain Medicine, Beth Israel Deaconess Medical Center, Harvard Medical School, Massachusetts, United States.; 6 Grupo de Bioingeniería y Telemedicina, Universidad Politécnica de Madrid - Madrid, Spain.; 7 Department of Radiology, Hospital Israelita Albert Einstein - São Paulo (SP), Brazil.

"Health is a right of all and an obligation of the State, guaranteed by socioeconomic policies that seek reduction of the risk of disease and of other grievances and universal and equal access to the actions and services in its promotion, protection and recuperation."

Constitution of the Federative Republic of Brazil, 1988

*Sistema Único de Saúde* (SUS) was established in 1990 by the Brazilian Federal Constitution to ensure comprehensive, universal and free access to healthcare for the entire population.^(1)^ In 1988, half of Brazil's population had no health coverage. Two decades after establishing SUS, more than 75% of the country's estimated 190 million people rely exclusively on SUS for their health care coverage.^(2)^ Provision of these services presents a rich opportunity to capture digital health data, which are a resource for developing locally relevant clinical practice guidelines rather than adopting those of other countries.

Despite this potential for data acquisition, electronic health records (EHR) in Brazil are currently used primarily to support administrative and billing functions and do not store clinical information in a machine-ready format as required for data analysis.^(3)^ A recent survey showed that 70% of the facilities that have used the Internet in the last 12 months in Brazil have some sort of electronic record for medical information.^(4)^ In 48% of the facilities, records are partially on paper and partially digital. Paperless information systems were present only in 22% of the facilities, and this rate was slightly higher at 33% for private facilities. On the other hand, 30% of the facilities keep all their patient records on paper with a much higher proportion among public facilities at 51%.^(4)^

Two government departments could play leading roles in moving health information technology and data analytics forward in Brazil: (1) DATASUS, which provides information systems support to all divisions of SUS,^(5)^ and (2) *Telessaúde Brasil Redes*, a national program designed to improve SUS's quality of care, integrating teaching and service through information technology tools.^(6)^ With the integration of SUS, it is possible to create an EHR for every citizen, a repository of an individual's records of services carried out and clinical data, and when aggregated, an accurate record of the trajectory of health and disease in Brazil.

Databases drawn from EHR in Brazil are limited in scope and accessible only to investigators internally within a hospital or organization. Indeed, two central factors prevent secondary analysis of health records in Brazil to improve population health: the lack of interoperability between information systems used in the health sector and the general attitude towards data as a resource. Although DATASUS provides open access to general government data to the public, these data do not currently constitute a research resource and are spread across systems.

However, it is important to emphasize some important initiatives in Brazil regarding EHR. *TBweb* is an online database that is used for epidemiological surveillance and monitoring of cases of tuberculosis in São Paulo state. It is a real-time system, and data can be uploaded and assessed online throughout the course of the disease.^(7)^ Additionally, *CadUnico* is a great example of an instrument that merges demographic characteristics, interventions and socioeconomic markers from different databases.^(8)^

In this scenario, initiatives promoting direct interaction between medical doctors, data scientists, managers and computer scientists with successful systems for Big Data Analytics could add practical value and foster future data integration in this country. We organized an event bringing together experts and a high-quality database for a hands-on experience in health data analytics. On May 6-7, 2017, a critical care datathon in Brazil was held at Hospital Israelita Albert Einstein (HIAE).

The term "datathon" is a portmanteau of data + hackathon, accentuating application of the hackathon model to data analytics. In this Brazilian edition, members of the Laboratory for Computational Physiology (LCP) at the Massachusetts Institute of Technology (MIT) provided guided access to Medical Information Mart for Intensive Care III (MIMIC-III), a well-curated open-access clinical database developed and maintained by LCP and supported by a vibrant research community.^(9)^ As has been the model for past datathons hosted by LCP, the MIT-HIAE event was split into two components over two days: a half-day conference on health informatics followed by a day and a half of hands-on analysis of clinical data.

The half-day health data conference was attended by 204 participants. Topics covered by presentations and panel discussions included the value of data sharing and multi-disciplinary collaboration around the data, privacy and security issues surrounding data sharing as it pertains to Brazil, and cultivation of an ecosystem of innovation around big health data and biotechnology. The critical care datathon immediately followed the conference. It was attended by 31 data scientists and 19 clinicians for a total of 50 attendees. Doctors pitched research questions that have been screened by the MIT and HIAE faculty to evaluate whether they could be addressed with the MIMIC database. These topics included the following: "day-night variation in sedation practices and patient outcomes", "mechanical power in mechanically ventilated patients", "the association between serum lactate trajectory and specific organ dysfunction", "effect of hyperoxia in patients with sepsis", "outcomes after surgery in very elderly patients admitted in the intensive care unit", "behavior of brain natriuretic peptide in acute kidney injury", and finally, "the impact of nighttime vs. daytime admissions on clinical outcomes."

Groups were formed based on expertise and interest in the particular clinical question. Early in the second and final day of the event, feedback sessions were held to gauge the progress of each group and provide suggestions regarding study design and additional analyses. At the end of the event, all teams gave a brief presentation of their research projects.

Bringing together clinicians and data scientists at the MIT-HIAE datathon served to demonstrate the value of each other's expertise. Most importantly, the datathon generated interest at HIAE to contribute to a high-resolution critical care database for the research community, supplementing existing resources, such as MIMIC. This decision might encourage other institutions in this country towards integrating and sharing medical databases in a standardized format, thus providing valuable insights to all health care ecosystems.

Change is on the horizon with growing interest in secondary analysis of health records to fuel a learning health system. However, for a true health data revolution to occur in Brazil, the environment - the technology, policies, and people, both providers and patients - needs to be supportive of change. Those at the forefront of the health data revolution must earn and maintain society's trust and demonstrate that data analysis benefits patients. The proponents of health data sharing tread a slippery path as they move into uncharted area. We hope that the datathon experience inspires similar initiatives in this country that will leverage data routinely collected in the process of care to inform clinical practice and health policies.
